# Comparative Study of Antibacterial, Antibiofilm, Antiswarming and Antiquorum Sensing Activities of *Origanum vulgare* Essential Oil and Terpinene-4-ol against Pathogenic Bacteria

**DOI:** 10.3390/life12101616

**Published:** 2022-10-17

**Authors:** Abderrahmen Merghni, Najla Haddaji, Nouha Bouali, Khulood Fahad Alabbosh, Mohd Adnan, Mejdi Snoussi, Emira Noumi

**Affiliations:** 1 Laboratory of Antimicrobial Resistance LR99ES09, Faculty of Medicine of Tunis, University of Tunis El Manar, Tunis 1007, Tunisia; 2 Department of Biology, College of Science, University of Hail, Hail 2440, Saudi Arabia; 3 Laboratory of Analysis, Treatment and Valorization of Environmental and Product Pollutants Faculty of Pharmacy of Monastir, Avicenne Street, Monastir 5000, Tunisia; 4 Laboratory of Genetics, Biodiversity and Valorisation of Bioresources, High Institute of Biotechnology, University of Monastir, Monastir 5000, Tunisia

**Keywords:** biofilm, *Origanum vulgare*, terpinene-4-ol, antibacterial, antiquorum sensing

## Abstract

Essential oils from aromatic and medicinal plants have many bioactive compounds known for their important biological activities mainly their antibacterial effects. Here we evaluated qualitatively and quantitatively the biofilm formation capability of pathogenic bacterial strains (n = 8). Then, we investigated the antibacterial, antibiofilm, antiquorum-sensing, and antiswarming efficacy of *Origanum vulgare* essential oil (EO) and terpinene-4-ol. Our results revealed that EO exhibited a more potent inhibitory effect against the tested strains. While the terpinene-4-ol was found to be more effective against developed *Staphylococcus aureus* biofilm. Regarding the anti quorum-sensing activity, we noticed that *O. vulgare* displayed better inhibition percentages in violacein production even at a low concentration (MIC/4). Additionally, this EO showed better inhibition of *Pseudomonas aeruginosa* PAO1 migration in comparison with the terpinene-4-ol. Our findings revealed that using pure *O. vulgare* EO demonstrated better competitive effects against pathogenic bacteria with a different mode of action when compared to the terpinene-4-ol. Hence, exploration and development of efficient anti-infection agents from natural resources such as full EOs represent promising tools in anti-infective therapy.

## 1. Introduction

Pathogenic bacteria are characterized by their ability to produce several virulence factors. One example is, biofilm formation, which provides them protection against the host immune system defense and allows for the acquisition of significant resistance to various antimicrobials [1]. This form of subsistence, well spread in natural ecosystems as well as in medical and industrial area, afford a solid inter-bacterial contact [2]. Numerous pathogenic bacteria ensure the control and monitoring of biofilm formation under a bacterial communication system known as quorum sensing (QS) [3]. In fact, numerous pathogenic bacteria have the ability to control the expression of their virulence factors through the QS system [4], along with the production of secondary metabolites and stress adaptation mechanisms [5].

One of the recent approaches in antibiotic therapy and biofilm dispersal is to target the bacterial QS system [6]. Therefore, developing innovative therapeutic measures based on novel antibiofilm agents with anti-QS properties are needed. Essential oils (EOs), well known for their bioactive compounds, have previously been proven to be effective in eradicating a wide range of pathogenic microorganisms [7]. *Origanum vulgare* L. EO has been extensively studied for its strong activity against many types of microorganisms due to the presence of the terpinene-4-ol, an oxygenated monoterpene [8,9,10]. This compound has strong antibacterial activity against several Gram-positive and negative species such as *Staphylococcus aureus*, *Bacillus subtilis*, *Enterococcus faecalis*, *Escherichia coli*, *Pseudomonas aeruginosa*, and other pathogenic microorganisms [11,12].

The present study aims to assess the biofilm formation ability of various pathogenic bacteria and to compare the antibacterial and antivirulence effects of O. vulgare EO and its main compound (terpinene-4-ol) against the studied bacteria.

## 2. Materials and Methods

### 2.1. Chemical Composition Analysis (GC–EIMS)

GC-EIMS analyses were performed according to the protocols previously described by Davies (1990) and Adams (1995) based on the calculation of the retention times using the n-alkanes series (C8–C23) [13,14].

### 2.2. Tested Agents and Microorganisms

The EO of *O. vulgare* and the terpinene-4-ol were purchased from Huiles & Sens (Entrechaux, France). Reference strains (*Chromobacterium violaceum* ATCC 12472, *Pseudomonas aeruginosa* PAO1, *Escherichia coli* ATCC 35218, *Salmonella enterica* CECT 443, *Shigella flexeneri* CECT 4804, *Staphylococcus aureus* ATCC 6538, *Bacillus subtilis* CIP 5265, *Vibrio vulnificus* CECT 529, and *Listeria monocytogenes* CECT 933) were procured from American Type Culture Collection (ATCC, Virginia, USA), Spanish Type Culture Collection (CECT, Valencia, Spain), and Institute Pasteur Collection (CIP, Paris, France). 

### 2.3. Disk Diffusion Assay

The activity of *O. vulgare* EO and Terpinene-4-ol was evaluated against four Gram negative (*P. aeruginosa* PAO1, *E. coli* ATCC 35218, *S. enterica* CECT 443, *S. flexeneri* CECT 4804) and four Gram-positive (*S. aureus* ATCC 6538, *B. subtilis* CIP 5265, *V. vulnificus* CECT 529, and *L. monocytogenes* CECT 933) bacteria, following a standard agar disk diffusion assay [15]. An inoculum of 0.5 McFarland was prepared for each strain and then swabbed onto the surface of Muller Hinton (MH, Bio-rad, Marnes-la-Coquette, France) agar. Whatman discs (Bio-rad, Marnes-la-Coquette, France) were impregnated with 10 μL of the EO and the compound, and then placed onto the surface of inoculated plates (Bio-rad, Marnes-la-Coquette, France). Gentamicin discs (Bio-rad, Marnes-la-Coquette, France) were used as a positive control. After 24 h of incubation at 37 °C, the diameters of inhibition zones (ZOI, in mm) were measured. All experiments were repeated three times.

### 2.4. Minimum Inhibitory and Minimum Bactericidal Concentrations 

The minimum inhibitory concentration (MIC) values for *O. vulgare* EO and terpinene-4-ol against each strain were determined by the broth dilution method [16]. All pathogens were cultured for 24 h and their optical density was adjusted to 0.5 McFarland standards. The tested agents were transferred to sterile 96-well microtiter plates. The inoculum (10 μL) of each strain was added to each well. MIC was defined as the concentration that completely inhibited visible cell growth during a 24 h incubation period at 37 °C. To determine the minimum bactericidal concentration (MBC) values, 10μL of each well medium with no visible growth was removed and pour plated with MH agar. After 24h of incubation at 37 °C, the number of surviving organisms was determined as CFU/mL [17].

### 2.5. Biofilm Formation Ability 

The ability to produce slime was carried out by culturing each bacterium on Congo Red Agar (CRA) as previously described [18]. After aerobic incubation at 37 °C for 24 h, strains with black colonies were identified as slime positive, while red colonies bacteria were classified as slime-negative strains [18].

Qualitative biofilm formation on glass surfaces was determined according to the protocol previously described by Davenport et al. (1986) [19]. Each strain was tested at least three times and biofilm production by each isolate was interpreted as negative, weak (1+), moderate (2+), or strong (3+). 

Quantitative biofilm production by tested strains was assessed using crystal violet staining assay as described previously [20]. Overnight bacterial culture grown in BHI (Bio-Rad, Marnes-la-Coquette, France) were diluted (1:100) in BHI with 2% glucose (*w*/*v,* (Bio-rad, Marnes-la-Coquette, France) and then incubated on 96-well tissue culture plates (Nunc, Roskilde, Denmark) for 24h at 37 °C. Adherent bacteria were stained with 1% crystal violet (Merck, Paris, France) for 5 min after their fixation with 95% ethanol (Bio-rad, Marnes-la-Coquette, France). The optical density of each well of the dry microplates was measured at 570 nm (OD_570_) using an automated reader (Bio-rad, Marnes-la-Coquette, France) and the biofilm formation was interpreted as highly positive (OD_570_ ≥ 1), low positive (0.1 ≤ OD_570_ < 1), and negative (OD_570_ < 0.1).

### 2.6. Antiadhesion Effect

The antiadhesion properties of *O. vulgare* EO and terpinene-4-ol were tested according to the protocol of Saising et al. [21]. A 100 μL aliquot of the bacterial growth in BHI supplemented with 2% glucose was transferred to a microtiter plate (Bio-rad, Marnes-la-Coquette, France) and added with 100 μL of different inhibitory concentration (1/16 to 1 × MIC) of the tested agents. After incubation for 24h at 37 °C, the supernatant was discarded and crystal violet (CV) stained biofilm cells were determined at 570nm using a microplate reader (Bio-rad, Marnes-la-Coquette, France). 

### 2.7. Reduction of Biofilms Growth and Development

The reduction in biofilms developed for 48 h at 37 °C by *O. vulgare* EO and terpinene-4-ol was evaluated as described previously [22]. A range of concentrations (1 × MIC, 2 × MIC, and 4 × MIC) of the selected agents were added to each well of the microplate per well and then incubated for 24 h. After treatment and staining (CV) assay, the biofilm biomass was measured by the absorbance of CV at 570 nm. The percentage of biofilm eradication was determined as:

[(OD growth control—OD sample)/OD growth control] × 100.

### 2.8. Antiquorum Sensing Activity

The qualitative analysis studied the reduction in violacein pigment. An overnight culture (10 μL) of *C. violaceum* (adjusted to 0.4 OD at 600 nm) was added into sterile microtiter plates containing 200 μL of LB broth and incubated at 30 °C in the presence and absence of various concentrations of tested agents (MIC = 10 mg/mL until MIC/32 = 0.3125 mg/mL). LB broth containing *C. violaceum* ATCC 12472 was used as a positive control [23]. The percentage of violacein reduction was calculated by following formula:

Violacein inhibition (%) = (OD_585 nm_ Control—OD_585 nm_ Sample)/OD_585 nm_ Control.

### 2.9. Antiswarming Activity

An overnight culture of *P. aeruginosa* PAO1 strain (5 μL, 0.4 OD at 600 nm) was utilized to inoculate the swarming agar medium (1% peptone, 0.5% NaCl, 0.5% agar, and 0.5% of filter-sterilized D-glucose) (Bio-rad, Marnes-la-Coquette, France) at three different concentrations of test agents (50, 75 and 100 μg/mL). The plates were incubated for 16 h at 30 °C. The decrease in swarming was interpreted by measuring the swarm zones of the bacterial cells after 16 h [24].

## 3. Results

### 3.1. Essential oil Composition

The chemical composition of *O. vulgare* EO is summarized in Table 1.

**Figure 1:**
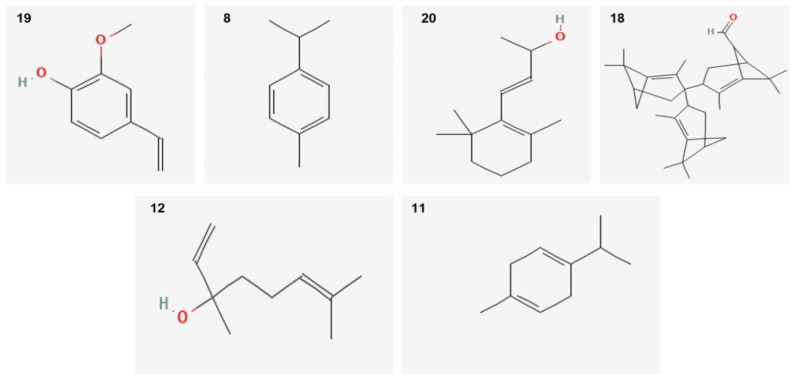
Sharper image structure of the main compounds identified in *O. vulgare* EO by GC-MS technique. Numbers are same listed in Table 1.

Twenty-two components with different percentages were identified using HP5 capillary column according to their elution time. *O. vulgare* EO was rich in Guaiacol–ρ-vinil (68.67%), p-Cymene (4.6%), and β-ionol (3.16%). Other relevant components were terpinen-7-ol (2.57%) and linalool (2.3%). The structures of the major compounds are represented in Figure 1.

### 3.2. Antibacterial Activity

Antibacterial effects are reported as inhibition zones, and in vitro activity as MIC and MBC. The obtained results using the disc diffusion method recorded in MH agar are summarized in Table 2. 

The EO of *O. vulgare* was active against all the tested strains with an inhibition zone ranging from 10.33 ± 0.57 mm to 41.66 ± 0.57 mm. Its zone of inhibition is larger to the size of the positive control antibiotic (Gentamicin) zone (Exception for *P. aeruginosa* and *S. aureus*). Terpinene-4-ol was found to be active again six pathogenic strains, with weak inhibition effect against *P. aeruginosa* PAO1 (less than 8 mm). However, no activity of this compound was observed against *B. subtilis* CIP 5265.

Considering the MIC and MBC values, both tested substances present a bacteriostatic effect against all tested pathogens at a concentration of 0.048 mg/mL. The MBC values of *O. vulgare* EO were found to be 1.562 mg/mL for all strains excepting *P. aeruginosa* PAO1 (50 mg/mL). The terpinene-4-ol exhibited MBC values weaker than the EO, ranging between 3.125 and 12 mg/mL (Table 2).

### 3.3. Biofilm Formation Activity

**Figure 2:**
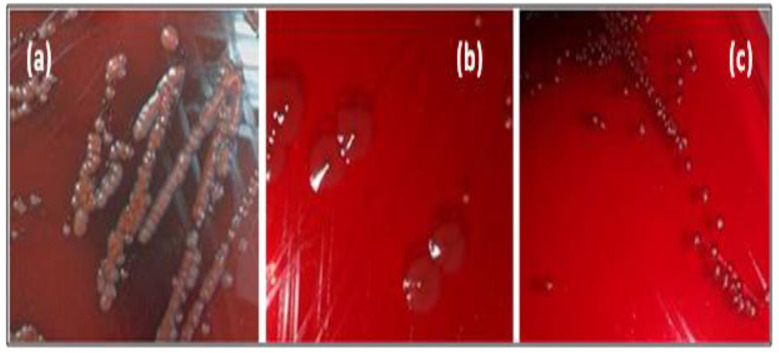
Different morphotypes of pathogenic strains cultivated on CRA. (**a**): negative morphotype, (**b**,**c**): positive morphotype.

Amongst tested strains, five out of eight bacterial pathogens (62.5%) displayed positive (black colony) and variable phenotype (black center) over CRA plates (Figure 2), indicating slime production (Table 3). 

Qualitative evaluation of biofilm formation potential on glass tube showed that only *S. aureus* were highly adherent (noted +++), five strains were moderately adherent (noted ++), and only two strains (*B. subtilis* and *S. enterica*) were weakly adherent (noted +, Table 3). The results of quantitative biofilm formation ability evaluated with CV staining assay revealed that among the tested bacteria, only *S. aureus* ATCC 6538 strain was highly biofilm positive (OD570 ≥ 1) over polystyrene surfaces (Table 3). Other strains showed low-grade biofilm formation (0.1 ≤ OD570 < 1). 

### 3.4. Antibiofilm Activity

**Figure 3:**
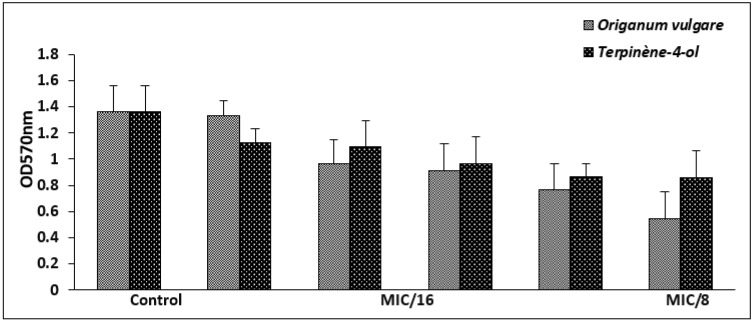
Biofilm formation of *S. aureus* ATCC 6538 in the presence of sub-inhibitory concentrations of *O. vulgare* EO and terpinene-4-ol. Error bars represent standard deviations.

The highly biofilm forming strain *S. aureus* ATCC 6538 was selected for the antibiofilm test. For antiadhesion assay, this bacterium was cultured in microtiter plates for 24 h in the presence of sub-inhibitory concentrations of the tested agents (1/16× to 1 × MIC) and the developed biofilm was stained with crystal violet. At a concentration of 1/8 × MIC, corresponding to 0.006 mg/mL against S. aureus ATCC 6538, the EO exerted an antiattachment effect (OD570 < 1), when compared to the control (untreated cells). However, the antiadhesion effect of the main compound was observed from at a concentration of 1/4 × MIC (Figure 3).

**Figure 4:**
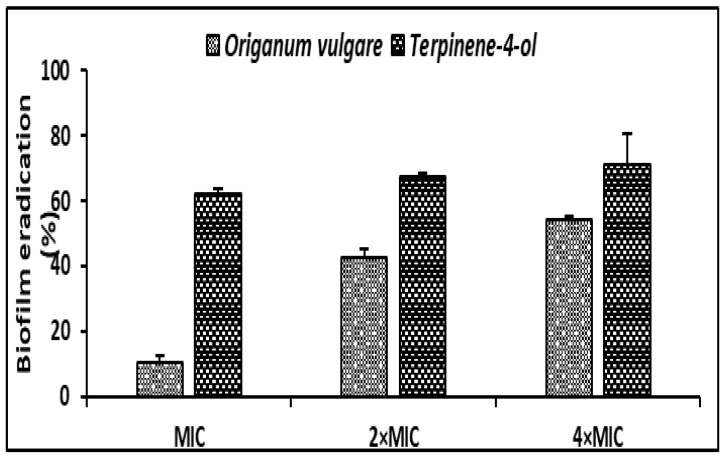
Antibiofilm effect of the *O. vulgare* EO and terpinene-4-ol against *S. aureus* ATCC 6538 using the Crystal Violet staining assay. Error bars represent standard deviations.

Regarding the antibiofilm effect of *O. vulgare* EO and the terpinene-4-ol, a mature biofilm (48h) of *S. aureus* was subjected to various concentrations (MIC, 2 × MIC, 4 × MIC) of the tested agents. *O. vulgare* EO and terpinene-4-ol were effective against the development of biofilms with percentage reduction values ranging from 10.36% ± 1.95 to 54.05% ± 1.48 and 62.28% ± 1.42 to 70.97% ± 9.65, respectively (Figure 4). We clearly noted that the terpinene-4-ol was more effective against *S. aureus* biofilm than the essential oil. 

### 3.5. Antiquorum Sensing Activity

In order to evaluate the extent of QSI, the violacein pigment production in *C. violaceum* ATCC 12472, in the absence or presence of *O. vulgare* EO and terpinene-4-ol at different concentrations was evaluated. Our results revealed that the EO inhibited the violacein production more efficiently, with observed percent inhibition of more than 50%, even at a low concentration (MIC/4). In contrast, terpinene-4-ol could inhibit violacein production only at high concentration (MIC) to an extent of 42.29±0.9 % (Table 4). 

### 3.6. Antiswarming Assay

As swarming migrations play an important role in QS-mediated biofilm formation in uro-pathogens, such as *P. aeruginosa* PAO1, we examined the anti-QS potential of *O. vulgare* EO and terpinene-4-ol against QS dependent swarming motility in this strain. The results obtained indicated that the tested agents inhibited the swarming behavior of the test PAO1 pathogen to different extents. In fact, the percentage of antiswarming activity of the main compound reached 25% independently of the concentration. Unlike the *O. vulgare* EO, which showed more inhibition level in the migration of PAO1 recording 29.17 ± 4.17% (Table 5).

## 4. Discussion

The QS is an efficient bacterial cell–cell communication process that controls numerous mechanisms, particularly in pathogenic bacteria, including antibiotic production, biofilm formation, and virulence factor secretion [4]. Recently, there has been a rise in attempts to search for and identify novel antimicrobials of natural origins to control the emergence of dreaded pathogens [25]. In the present study, we characterized the biofilm formation ability of eight pathogenic bacteria. Then, we investigated the antibacterial, antibiofilm, antiquorum-sensing, and antiswarming efficacy of *O. vulgare* essential oil and its main compound Terpinene-4-ol. 

The results of the first part of our study conducted on biofilm characterization, determined by different phenotypic assays, revealed that *S*. *aureus* strain exhibited the highest biofilm production capability when compared to the rest of the pathogenic bacteria. As largely documented in the literature, this bacterium is well known for its efficacy to colonize various biotic and abiotic surfaces [1,26]. Secretion of extracellular polymeric substance matrix by *S*. *aureus* leads to the establishment of a solid structure called microbial biofilm [27]. Such polymers surrounding the bacterial micro-colonies allow them protective effects against various external agents [28]. In fact, the resistance to antibiotics, disinfectants, and host defense systems increases when the bacteria are implicated into the biofilms [29]. Due to the continuous increase in bacterial infection rates related to microbial biofilm, the necessity to search and develop new active substances with low toxicity remain of interest.

In the second part of our investigation, we tested the antibacterial effects of *O. vulgare* EO and terpinene-4-ol. Observing the results of the agar disc diffusion method, it is possible to conclude that pure EO exhibited the highest inhibition zones against most of the tested strains when compared to the tested compound and the reference antibiotic (Gentamicin). Interestingly, the EO showed 6 times greater inhibitory effect with ZOI ≥ 28 mm [30] which is statistically significant (*p* ˂ 0.05) when compared to the terpinene-4-ol (4 times stronger inhibitory effect). Additionally, *O. vulgare* EO showed more efficient bacteriostatic impact against almost all tested strains with an MBC value of 1.562 mg/mL. The differences in the effects of natural substances can be due to the synergism between the minor and major molecules composing the *O. vulgare* EO, unlike the unique major compound [31,32]. Moreover, these differences might be attributed to the differences in the susceptibility of tested species, since the outer membrane of Gram-negative bacteria are characterized by the presence of lipopolysaccharide molecules, which provide a hydrophilic surface [33]. Accordingly, Gram-positive strains are relatively more sensitive to hydrophobic compounds such as EOs [34].

Since bacterial cell attachment represents a critical essential step in biofilm formation, we tested the effect of sub-inhibitory concentrations of the *O. vulgare* EO and Terpinene-4-ol against the adhesion of the selected strains. Our result demonstrated that at a concentration of 0.006 to 0.018 mg/mL, corresponding to 1/8 × MIC and 1/4 × MIC, respectively, the tested agents exerted an antiattachment effect against the highly biofilm forming strain (*S*. *aureus* 6538). The effects of EOs and their compounds against the adhesiveness of *S. aureus* have been frequently reported [22,35]. Interestingly, the use of EOs as natural antibacterial agents, for inhibition of cell attachment of pathogenic bacteria represents a strategic way to prevent the establishment and the development of mature resistant biofilm [36]. The result of biofilm eradication showed that terpinene-4-ol was more effective against the development of preformed *S. aureus* biofilm (*p* < 0.05), with a percentage of reduction values exceeding 60% even at a concentration of 1 × MIC. Our results are in agreement with previous findings recording the effectiveness of various main compounds from EOs such as 1,8 cineol, thymol, and carvacrol in the eradication of staphylococcal biofilms [35,37,38]. Additionally, the efficacy of EOs, as antibiofilm agents from natural origins, was previously documented against several Gram-positive and Gram-negative bacteria [39]. 

Biologically active substances that interact with bacterial QS systems attenuating their pathogenicity are known as anti-QS compounds. In this part of our study, we evaluated the anti-QS activity *O. vulgare* EO and terpinene-4-ol, using *C. violaceum* ATCC12472 as a biomonitor strain. From this test, it was observed that the EO presented better anti-QS activity (*p* ˂ 0.05) than the compound in a concentration-dependent manner. The inhibition effect of QS by *O. vulgare* EO from different origins was reported in previous studies [8,10]. The synergistic effects between all the compounds present in this EO leads to the inhibition of bacterial intercellular communication systems and subsequently affects the ability to form biofilms and produce virulence factors in pathogenic bacteria [40,41].

Bacterial swarming motility is one important virulence factor mediated and regulated by the QS system [42]. Our results revealed that a reduction in swarming motility of PAO1 by tested agents was observed in a different manner. In fact, the EO inhibits the migration of PAO1 at higher concentrations (*p* ˂ 0.05), whereas the effect of terpinene-4-ol is the same regardless of the concentration. It was reported that sub-MICs of various EOs such as tea tree, eucalyptus, and clove revealed a decrease in swarming motility in *P. aeruginosa* PAO1 in a concentration-dependent manner [35,38]. Interestingly, the inhibition of swarming migration is a promising strategy to fight against pathogenic bacteria since it represents one of the dreaded virulence factors involved in biofilm formation [24].

## 5. Conclusions

In summary, *O. vulgare* EO and terpinene-4-ol exhibit various inhibitory effects against pathogenic bacteria. Considering that *O. vulgare* EO has the highest antibacterial, antiquorum sensing, and antiswarming potential, it is therefore recommended to be valorized as an efficient antimicrobial agent for the treatment of bacterial infections. More investigations are also necessary to elucidate the biological activities of the main compounds identified in *O. vulgare* essential oil and their possible use in the industrial formulation of essential oil products. 

## Figures and Tables

**Table 1:** Essential oil composition (%) of *Origanum vulgare* essential oil.

N.	Compound	%	Ki	Kr
1	Sabinene	0.79	965	975
2	1-Octen-3-ol	0.20	971	979
3	3- Octanone	0.14	978	983
4	Myrcene	0.21	982	990
6	δ- 2-carene	0.11	994	1002
7	δ- 3-carene	0.47	1006	1011
8	*p*-Cymene	4.6	1015	1024
9	Limonene	0.82	1019	1029
10	1,8 cineole	1.23	1021	1031
11	γ-Terpinene	2.22	1050	1059
12	Linalool	2.30	1094	1096
13	Cis-Limonene oxide	0.13	1135	1136
14	Isoborneol	1.24	1158	1160
15	Terpinene-4-ol	0.44	1170	1171
16	α-Terpineol	0.47	1183	1188
17	Thymol methyl ether	0.17	1236	1235
18	Terpinen-7-ol	2.57	1286	1285
19	Guaiacol–ρ-vinil	68.67	1299	1309
20	β-ionol	3.16	1413	1414
21	α- Pachoulene	1.07	1446	1451
22	Spathulenol	0.68	1576	1578

Ki: Kovats retention index determined relative to the tR of a series of n-alkanes (C10–C35) on an HP-5 MS column; Kr: Kovats retention index determined relative to the tR of a series of n-alkanes (C10–C35) on HP Innowax.

**Table 2:** Antibacterial activity of *O. vulgare* EO and the terpinene-4-ol against pathogenic bacteria.

Strains	*Origanum vulgaris* EO			Terpinene-4-ol			Gentamicin IZ (mm ± SD)
	IZ (mm ± SD)	MIC (mg/mL)	MBC (mg/mL)	IZ (mm ± SD)	MIC (mg/mL)	MBC (mg/mL)	
CECT 933	41.66 ± 0.57	0.048	1.562	35 ± 0.89	0.048	3.125	26 ± 0.01
CECT 529	32.33 ± 0.57	0.048	1.562	21 ± 0.01	0.048	12.5	21 ± 0.01
CECT 4804	33.33 ± 0.99	0.048	1.562	40 ± 0.01	0.048	3.125	22 ± 0.01
CIP 5265	38.33 ± 0.89	0.048	1.562	6 ± 0.01	0.048	3.125	26 ± 0.01
CECT 443	36.66 ± 0.89	0.048	1.562	38 ± 0.01	0.048	3.125	28 ± 0.01
ATCC 35218	28.66 ± 0.57	0.048	1.562	25.5 ± 0.71	0.048	3.125	22 ± 0.01
PAO1	10.33 ± 0.57	0.048	50	7.33 ± 0.57	0.048	12.5	15 ± 0.01
ATCC 6538	27.33 ± 0.89	0.048	1.526	30 ± 0.01	0.048	3.125	32 ± 0.01

IZ: Inhibition zone; SD: Standard deviation. Gentamicin concentration= 10 mg/mL. CECT 933: *L. monocytogenes*; CECT 529: *V. vulnificus*; CECT 4804: *S. flexeneri*; CIP 5265: *B. subtilis* CECT 443: *S. enterica* ATCC 35218: *E. coli*; PAO1: *P. aeruginosa;* ATCC 6538: *S. aureus.*

**Table 3:** Slime production and adhesive properties of selected pathogenic strains.

Strains	Adhesion to Glass	Slime Production on CRA		Adhesion to Polystyrene	
		Color	S+/S−	OD_570_ ± SD	Production of Biofilm
*S*. *aureus* ATCC 6538	+++	Black	S+	1.36 ± 0.20	High production
*P. aeruginosa* PAO1	++	Red border	S−	0.42 ± 0.26	Low production
*E*. *coli* ATCC 35218	++	Red with black center	S+	0.17 ± 0.03	Low production
*S. flexeneri* CECT 4804	++	Red with black center	S+	0.10 ± 0.01	Low production
*B*. *subtilis* CIP 5265	+	Red border	S−	0.12 ± 0.01	Low production
*V*. *vulnificus* CECT 529	++	Red with black center	S+	0.13 ± 0.02	Low production
*S*. *enterica* CECT 443	+	Red border	S−	0.15 ± 0.01	Low production
*L*. *monocytogenes* CECT 933	++	Red with black center	S+	0.19 ± 0.07	Low production

OD: Optical density; SD: Standard deviation; +: low adhesion; ++: moderate adhesion; +++: High adhesion; S+: Slime producer; S−: Non slime producer.

**Table 4:** Percentage of violacein inhibition using *C. violaceum* ATCC 12472 strain.

Concentration	% of Violacein Inhibition	
	*O. vulgare*	Terpinene-4-ol
MIC	72.7± 1.5	42.29 ± 0.9
MIC/2	67.87 ± 1.7	32.17 ± 1.2
MIC/4	53.74 ± 0.78	28.14 ± 1.1
MIC/8	37.52 ± 1.1	16.98 ± 1.4
MIC/16	30.34 ± 1.3	8.52 ± 0.5
MIC/32	14.88 ± 0.7	4.68± 1.1

**Table 5:** Effect of *O. vulgare* EO and terpinene-4-ol on swarming motility of PAO1.

Component	Concentration (µg/mL)		
	50	75	100
	% of Swarming Motility Inhibition		
*O. vulgare*	25 ± 8.33	25 ± 0.01	29.17 ± 4.17
Terpinene-4-ol	25 ± 0.01	25 ± 0.01	25 ± 0.01

## Data Availability

Not applicable.
